# Correlates of high-dose antipsychotic prescription amongst outpatients with Schizophrenia in a Nigerian Hospital

**DOI:** 10.4102/sajpsychiatry.v28i0.1791

**Published:** 2022-04-29

**Authors:** Ihechiluru G. Anozie, Bawo O. James, Joyce O. Omoaregba, Sunday O. Oriji, Paul O. Erohubie, Anthony C. Enebe

**Affiliations:** 1 Department of Clinical Sciences, Federal Neuropsychiatric Hospital, Benin City, Nigeria; 2 Department of Mental Health, Nnamdi Azikiwe University, Awka, Nigeria; 3 Department of Mental Health, Irrua Specialist Teaching Hospital, Irrua, Nigeria; 4 Department of Mental Health Services, Federal Medical Centre Asaba, Asaba, Nigeria

**Keywords:** high dose, antipsychotics, prescription, schizophrenia, Nigeria

## Abstract

**Background:**

Treatment guidelines recommend the use of antipsychotic monotherapy at effective doses for the treatment of schizophrenia, although about a third of the sufferers still receive high-dose antipsychotic treatment. Current evidence suggests that high-dose antipsychotic prescription (HDAP) not only fails to improve outcomes but also increases side effects.

**Aim:**

Our study aimed to determine the prevalence of HDAP and its association with illness severity, medication adherence behaviour and side effects amongst outpatients with schizophrenia.

**Setting:**

The Federal Neuro-Psychiatric Hospital, Benin-City, Nigeria.

**Methods:**

A cross-sectional study of 320 attendees with schizophrenia at the outpatient department was undertaken. We administered a sociodemographic and antipsychotic medication questionnaire, Mini-International Neuropsychiatric Interview, Positive and Negative Syndrome Scale, Liverpool University Neuroleptic Side Effects Rating Scales and Medication Adherence Rating Scales. High-dose antipsychotic prescription was determined by the ratio of prescribed daily dose to defined daily dose greater than 1.5.

**Results:**

The prevalence of HDAP was 38.4%. Greater severity of illness, experiencing more side effects and poor medication adherence were significantly associated with HDAP.The major predictors of HDAP were antipsychotic polypharmacy and concurrent anticholinergic use.

**Conclusion:**

We conclude that although the use of HDAP amongst patients with schizophrenia remains common, its persistent use should be discouraged.

## Introduction

Schizophrenia is a clinical syndrome of variable, but profoundly disruptive psychopathology that affects cognition, emotion, perception and other aspects of behaviour.^1^ The associated burden of disease is such that its core symptoms, comorbid conditions or pharmacotherapy may result in social and occupational dysfunction in affected individuals and may reduce interpersonal functioning, decline in educational attainment, independent living skills and quality of life.^2^ Antipsychotics, grouped into typical and atypical antipsychotics are psychotropic agents used mainly in the treatment of schizophrenia. Although the complete mechanism of action of antipsychotics remains unclear, they exert their effects through central dopamine and in newer agents possibly via serotonergic antagonism.^3,4^ They also affect a number of other receptors in the brain, resulting in a wide range of unwanted effects.

In first episode psychosis, there is usually a remarkable response to antipsychotics. However, evidence has shown that up to a third of patients with chronic schizophrenia do not respond to medication, resulting in treatment resistance. This is defined as a lack of optimal response to trials of at least two different classes of antipsychotics within at least a 16-week span.^5^ In an effort to improve outcomes, the empirical use of high antipsychotic doses or combining two or more antipsychotics appeared to be adopted by clinicians, giving rise to the concept of ‘high dose’ antipsychotic treatment.^6,7^

Current evidence suggests that ‘high dose’ antipsychotic prescription do not improve the overall clinical outcomes in poor responders, but results in exacerbation of side effects.^8^ Despite the lack of scientific evidence to support its effectiveness, the global practice of prescribing high doses of antipsychotics sadly remains common amongst psychiatrists.^9^ There is also a lack of evidence-based research on high-dose antipsychotic prescription and its correlates in sub-Saharan Africa at large and Nigeria in particular. Consequently, our aim was to determine the prevalence of high-dose antipsychotic treatment and access its association with illness severity, medication adherence behaviour and side effects amongst outpatients with schizophrenia at the Federal Neuro-Psychiatric Hospital, Benin-City, Nigeria.

## Methods

### Study design and location

It was a descriptive, cross-sectional study carried out at the Out-Patient Department of the Federal Neuro-Psychiatric Hospital (FNPH), Benin-City, Nigeria. The hospital is a 270-bed facility that provides mental healthcare and general medical services to the South-South region of Nigeria.

### Study participants

Study population comprised 320 participants within the age range of 18–65, who had a diagnosis of schizophrenia made by a consultant psychiatrist in line with the 10th edition of the International Classification of Diseases (ICD-10) diagnostic criteria and had willingly given a written informed consent for their participation in the research. Service users who were uncooperative at the time of recruitment or had received antipsychotic medications for less than 6 months at the time were excluded from our study.

### Sample size calculation and sampling method

Based on the prevalence of 38% obtained in a similar study,^10^ the sample size of our was calculated and adjusted for a population of less than 10 000, with an inflationary rate of 10% added to increase the power of our study, resulting in a total of 320 participants. A systematic random sampling technique was adopted.

### A working definition of high-dose antipsychotic prescription

The operational criteria for high-dose antipsychotic treatment in our study was determined as the ratio of prescribed daily dose (PDD) to defined daily dose (DDD) greater than 1.5 as was used in various other studies.^10,11,12,13^ The DDD antipsychotics values were determined by the World Health Organisation (WHO) Collaboration Centre for Drug Statistics Methodology based on the doses used for the treatment of psychosis and were approved by WHO in 2003.^14^ For depot injections, the DDDs were calculated by dividing the prescribed dose with the dosing interval. In cases of polypharmacy, a PDD/DDD ratio was calculated as the sum of the individual PDD/DDD ratios of all antipsychotics prescribed to each affected patient.

### Instruments

#### Sociodemographic questionnaire

The authors designed a questionnaire that was used to capture data on important demographic and clinical variables of the participants such as age, gender, employment, educational status, marital status, duration of illness, duration of treatments and cost of medication.

#### Antipsychotic medication and health questionnaire

This questionnaire was specifically developed to obtain essential data on the prescription of antipsychotics medications (oral and depot antipsychotics), its dosing schedule, the PDD of antipsychotics and use of anticholinergic agents.

#### The mini-international neuropsychiatric interview version 6.0 (psychosis module)

The Mini-International Neuropsychiatric Interview (M.I.N.I.), a structured diagnostic interview schedule, was used in the confirmation of the diagnosis of schizophrenia.^15^

#### Positive and negative syndrome scale

The positive and negative syndrome scale (PANSS) comprises three subscales, which are the positive, negative and general psychopathology subscales. It was used to determine the pattern of psychopathology and illness severity of respondents.^16^

#### Liverpool University neuroleptic side effects rating scales

The Liverpool University Neuroleptic Side Effects Rating Scale (LUNSERS) was used to assess the side effects of antipsychotic drugs.^17^ Higher LUNSERS score indicates more side effects.

#### Medication adherence rating scale

Medication Adherence Rating Scale (MARS) was used to assess medication adherence amongst the respondents.^18^ Total scores on MARS range from 0 to 10 with a higher score indicating better medication adherence.

### Data analysis

The Statistical Package for the Social Sciences, version 23 (IBM SPSS version 23.0 Armonk, NY: IBM Corp.) was used to analyse the collated data and results presented in tables. The outcome variable used for bivariate analysis was the proportion of patients on high dose antipsychotic prescription (HDAP). Chi-square test and student’s *t*-test were used to test the associations between categorical and continuous variables and HDAP, respectively. A multivariate test in which significant associations on bivariate analysis were analysed with HDAP as the dependent variable was performed to identify its independent predictors. Statistical significance was set at *p* < 0.05.

### Ethical considerations

The Ethics and Research Committee of the FNPH, Benin-City, Edo State, Nigeria, on 11 November, 2016 (Protocol Number: PH/A.864/Vol.X/55) granted the approval for our study.

## Results

### Sociodemographic and clinical characteristics of participants

The mean (standard deviation [SD]) age of the participants was 37.13 (10.11) years, while the most common age class was 31–40 years. There were 153 (47.8%) males and 167 (52.2%) females, in which two-thirds (68.1%) of the participants were classed as ‘single’. Only a third of the participants (*n* = 112, 35%) were employed, with 45.5% of the employed earning the monthly wage of $50 or below.

Using the ICD-10 diagnostic criteria, 72.5% of the participants had a diagnosis of paranoid schizophrenia, and nearly half of the subjects (49.7%) had a current psychotic episode. The mean (SD) duration of illness of the study participants was 114.00 (82.80) months and the mean (SD) duration of treatment of 89.93 (77.26) months. The mean (SD) duration of untreated psychosis was 24.07 (36.36) months with a range of 1–225 months.

### Prevalence of high dose prescribing

Using the DDD convention of the ratio of PDD/DDD greater than 1.5, the prevalence rate of high-dose antipsychotic prescribing was 38.4%. This was higher when compared with prevalence rates of 8.1% and 2.2% obtained using British National Formulary (BNF) percentage exceeding 100% and chlorpromazine equivalents (CPZEq) > 1000 mg, respectively (Table 1).

**TABLE 1:** Prevalence of high dose prescribing.

Variable	Frequency	Percentage
**High dose (PDD/DDD)**		
Yes	123	38.4
No	197	61.6
**High dose (BNF %)**		
Yes	26	8.1
No	294	91.9
**High dose (CPZEq)**		
Yes	7	2.2
No	313	97.8

PDD, prescribed daily dose; DDD, defined daily dose; CPZEq, chlorpromazine equivalent; BNF, British National Formulary.

PDD/DDD: Prescribed daily dose/defined daily dose > 1.5

British National Formulary > 100%

CPZEq: Chlorpromazine equivalent > 1000 mg

### Association between sociodemographic variables of participants and high dose prescribing

As shown in Table 2, males were significantly more likely to receive HDAP compared with females [*p* = 0.03; odds ratio (OR) (95% confidence interval ([CI] = 1.63 [1.01–2.63]). Participants who were classed as single, separated, divorced or widowed were 2.5 times more likely to be on a HDAP compared with those who were married (*p* = 0.003, OR [95% CI] = 2.52 [1.31–5.07]). Also, unemployment status indicated a nearly two-fold increased risk of being on ‘high dose’ antipsychotic treatment compared with those employed (*p* = 0.01, OR [95% CI]: 1.94 [1.16–3.30]).

**TABLE 2:** Comparison of sociodemographic characteristics of participants and high dose prescribing.

Variable	High dose				Statistics			
	Yes		No		*X* ^2^	OR	95%CI	*P*
	*n*	%	*n*	%				
**Gender**	-	-	-	-	4.47	1.63	1.01–2.63	0.030
Male	68	55.3	85	43.1	-	-	-	-
Female	55	44.7	112	56.9	-	-	-	-
**Marital status**	-	-	-	-	8.65	2.52	1.31–5.07	0.003
Single, separated,	108	87.8	146	74.1	-	-	-	-
widowed or divorced	-	-	-	-	-	-	-	-
Married	15	12.2	51	25.9	-	-	-	-
**Employment status**	-	-	-	-	7.10	1.94	1.16–3.30	0.010
Unemployed	91	74.0	117	59.4	-	-	-	-
Employed	32	26.0	80	40.6	-	-	-	-

CI, confidence interval; OR, odds ratio.

Participants on HDAP spent significantly more money on medications monthly (*t* = 3.31, *p* < 0.001) had a longer duration of treatment (*t* = 2.78, *p* = 0.003) and longer duration of illness (*t* = 2.96, *p* = 0.01) (Table 3).

**TABLE 3:** Comparison of sociodemographic characteristics (continuous variables) of participants and high dose prescribing.

Variable	High dose				Statistics	
	Yes		No		*t*	*P*
	Mean	SD	Mean	SD		
Age	36.46	9.50	37.56	10.48	−0.95	0.340
Average medication cost	3787.40	3132.10	2785.30	2274.10	3.31	0.001
Duration of treatment (months)	104.99	73.21	80.53	78.41	2.78	0.003
Duration of illness (months)	131.11	78.31	103.32	83.93	2.96	0.010
Duration of untreated psychosis (months)	26.11	34.51	22.79	37.49	0.80	0.430

SD, standard deviation.

### Association between clinical-related characteristics of participants and high dose prescribing

Participants on polypharmacy (*p* < 0.001, OR [95% CI]: 29.15[14.07–64.17]), an anticholinergic medication (*p* < 0.001, OR [95% CI]: 12.87 [7.19–23.23]) and those diagnosed as having a ‘current psychotic episode’ on the MINI (*p* < 0.001, OR [95% CI]:6.74[3.93–13.84]), were found to have higher odds of being on HDAP. Other important clinical variables such as hypertension, alcohol use and use of other psychoactive substances, BMI, use of antidepressants use, use of mood stabilisers or oral hypoglycaemic agents did not show any significant association with HDAP (Table 4).

**TABLE 4:** Comparison of clinical related characteristics of participants and high dose prescribing.

Variable	High dose				Statistics			
	Yes		No		*X* ^2^	OR	95%CI	*p*
	*n*	%	*n*	%				
**Antipsychotic therapy**	-	-	-	-	126.680	29.15	14.07–64.17	-
Polypharmacy	112	91.1	51	25.9	-	-	-	0.001
Monotherapy	11	8.9	146	74.1	-	-	-	-
**Anticholinergic use**	-	-	-	-	-	-	7.19–23.23	-
Yes	98	79.7	46	23.4	97.060	12.87	-	0.001
No	25	20.3	151	76.6	-	-	-	-
**Alcohol use**								
Yes	11	8.9	10	5.1	1.850	-	-	0.170
No	112	91.1	187	94.9	-	-	-	-
**Other psychoactive substance use**								
Yes	12	9.8	19	9.6	0.001	-	-	0.970
No	111	90.2	178	90.4	-	-	-	-
**History of hypertension**								
Yes	6	4.9	17	8.6	1.600	-	-	0.210
No	117	95.1	180	91.4	-	-	-	-
**BMI**								
Underweight/Normal	78	63.5	124	62.9	0.010	-	-	0.930
Overweight/Obese	45	36.5	73	37.1	-	-	-	-
**Antihypertensive use**	-	-	-	-	2.460	-	-	0.120
Yes	5	4.1	17	8.6	-	-	-	-
No	118	95.9	180	91.4	-	-	-	-
**Lipid-lowering agent use**								
Yes	-		1	0.5	-	-	-	1.000*
No	123	100.0	196	99.5	-	-	-	
**Antidepressant use**								
Yes	3	2.4	11	5.6	1.790	-	-	0.180
No	120	97.6	186	94.4		-	-	
**Mood stabilizer use**								
Yes	1	0.8	2	1.0	0.030	-	-	0.860
No	122	99.2	195	99.0	-	-	-	-
**Oral hypoglycaemic use**								
Yes	3	2.4	4	2.0	0.060	-	-	0.810
No	120	97.6	193	98.0	-	-	-	
**MINI Diagnosis**	-	-	-	-	-	-	3.93–13.84	0.001
Psychotic disorder (current)	94	76.4	64	32.5	58.480	6.74	-	-
Psychotic disorder (lifetime)	29	23.6	133	67.5	-	-	-	-

BMI, body mass index; MINI, Mini-International Neuropsychiatric Interview.

* , Fisher’s Exact Test.

### Association between severity of psychopathology, side effects and medication adherence scores of participants and high dose prescribing

Respondents on HDAP reported significantly higher mean PANSS total scores (*t* = 9.26, *p* < 0.001) and on the three sub-scales. Participants receiving HDAP were significantly more likely to report side effects (*t* = 5.63, *p* < 0.001). In particular, they were more likely to report psychic, extrapyramidal, other autonomic, hormonal and anticholinergic side effects. In addition, participants on HDAP were significantly less likely to be adherent to medications (*t* = -6.55, *p* < 0.001) (Table 5).

**TABLE 5:** Comparison of Liverpool University neuroleptic side effects rating scale and medication adherence rating scale scores of participants and ‘high dose’ prescribing.

Variable	High dose				Statistics	
	Yes		No		*t*	*p*
	Mean	SD	Mean	SD		
LUNSERS total score	11.72	6.17	7.49	6.77	5.63	0.001
LUNSERS allergic	0.06	0.32	0.08	0.47	−0.40	0.690
LUNSERS psychic	3.76	2.75	2.50	2.91	3.82	0.001
LUNSERS EPSEs	3.95	3.77	1.88	2.74	5.68	0.001
LUNSERS other autonomic	0.77	1.23	0.33	0.87	3.76	0.040
LUNSERS hormonal	0.68	1.20	1.20	2.50	−2.03	0.001
LUNSERS anticholinergic	1.57	1.81	0.73	1.43	4.59	0.001
LUNSERS miscellaneous	0.89	1.36	0.77	1.16	0.84	0.400
MARS	6.27	2.45	7.96	2.12	−6.55	0.001

LUNSERS, Liverpool University Neuroleptic Side Effects Rating Scale; MARS, Medication Adherence Rating Scale; EPSEs, extra-pyramidal side effects; SD, standard deviation.

### Predictors of high-dose antipsychotic prescribing

When a binary logistic regression analysis was performed with the significant variables from the bivariate testing entered into the model using a backward conditional model, receiving HDAP was strongly predicted by polypharmacy (adjusted odds ratio [aOR] [95% CI]: 13.12 [5.12–33.58], *p* < 0.001), and anticholinergic use (aOR [95% CI]: 3.66 [1.53–8.73], *p* = 0.003]). In addition, current psychotic episode (*p* = 0.04), higher average cost of medication (*p* < 0.001), longer duration of treatment (*p* = 0.01) and hormonal side effects (*p* = 0.03) were also found to ‘predict’ high-dose antipsychotic treatment (Table 6).

**TABLE 6:** Independent predictors of ‘high dose’ prescribing.

Variable	B	SE	Wald	OR	95%CI	*p*
**Nature of therapy**	2.57	0.480	28.80	-	-	0.001
Monotherapy	-	-	-	1	Ref	-
Polypharmacy	-	-	-	13.12	5.12–33.58	-
**Anticholinergic use**	1.30	0.440	8.54	-	-	0.003
No	-	-	-	1	Ref	-
Yes	-	-	-	3.66	1.53–8.73	-
**MINI diagnosis**	−1.03	0.510	4.06	-	-	0.040
**Average monthly cost of medication**	0.00	0.000	13.68	-	-	0.001
**Duration of treatment**	−0.01	0.002	7.35	-	-	0.010
**LUNSERS Hormonal side effects**	0.19	0.090	4.67	-	-	0.030
**Constant**	2.02	0.650	9.64	-	-	-

LUNSERS, Liverpool University Neuroleptic Side Effects Rating Scale; CI, confidence interval; SE, standard error; OR, odds ratio; MINI, Mini-International Neuropsychiatric Interview.

## Discussion

### Sociodemographic characteristics of participants

Females (*n* = 167) were slightly more represented than their male counterparts in our study. This could be a reflection of the eagerness of females to attend scheduled visits compared with men. Conversely, more males are represented in inpatient population studies, especially when participants were recruited across all available diagnostic groups.^10,19,20^

A total of 8 in 10 (82.5%) participants had some form of secondary education. Other researchers also reported similar findings in this locality.^10,21^ However, the level of education achieved by the participants did not seem to translate into regular employment, as only a third (35%) of participants had some form of occupation mainly in ‘low skill’ jobs as service and sales workers or in elementary occupations as labourers. Other relevant research findings reported employment rates range of 26% – 44% amongst patients with schizophrenia in Nigeria,^10,21,22^ which also compares with the rate of 10% – 25% in developed nations amongst patients with schizophrenia.^23,24^

According to the report of the National Bureau of Statistics, the unemployment rate in Nigeria has risen to an all-time high of 23.1% in the third quarter of 2018.^25^ Furthermore, due to stigma, social exclusion and prejudicial treatment meted on persons living with mental illness, their rates of unemployment are far higher than that of the general population.^26^ Factors implicated in the rising rates of unemployment amongst persons living with mental illness include poor budgetary funding for mental healthcare and administration in Nigeria, a lack of ready availability of newer psychotropic agents, marked absence of specific social welfare and employment schemes such as sheltered employment and ‘individual placement and support’ targeted at persons living with schizophrenia.^27^ Sadly, about half (45.5%) of employed participants earned below the national minimum wage bracket of $50 per month as on 2018, indicating the poor quality of life of subjects in this environment.

### Prevalence of high-dose antipsychotic prescription

Using the operational definition of HDAP for our study, the prevalence obtained in our study was 38.4%. Similar findings were reported by Adesola et al. in Nigeria, who performed an inpatient hospital audit for high dose prescribing across all diagnostic groups^10^ suggesting that the practice of HDAP may not have changed amongst psychiatrists in the locality. Probable reasons could be that the severity of illness alone as seen in inpatient cohorts may not be the only rationale behind the use of high dose prescribing by clinicians, as it may be utilised in the chemical control of agitated patients in the acute phase of care. Other possible factors may include the reluctance of prescribers to change, as well as the lack of locally adapted treatment guidelines in the country.

When BNF percentage maximum exceeding 100% and CPZEq > 1000 mg were used as the criteria for the determination of high dose prescribing, the prevalence of HDAP was 8.1% and 2.2%, respectively. This infers that multiples of DDD criteria could be a better comparator for high dose determination than the CPZEq and BNF% maximum criteria. Therefore, although CPZEq has proven useful in clinical practice, its value in the determination of HDAP may be limited as equivalent values were derived empirically from expert consensus documents while considering its inappropriateness for dose equivalence with atypical antipsychotics.^28,29,30^ Also, the operational criteria was preferred to BNF% maximum since DDD values were calculated based on published systematic reviews and not with recourse to the formulary of the United Kingdom.^28^ For instance, the BNF had no maximum licensed dose for the trifluoperazine, with an arbitrary daily limit of 30 mg – 50 mg, making high dose determination difficult in a way that it could constitute a measurement bias in our study, given that trifluoperazine was a commonly used typical antipsychotic in our study location.^31^ Furthermore, the BNF maximum licensed dose for risperidone is 16 mg, despite the fact that its near-maximal effective dose (i.e. the threshold dose eliciting clinical response with the least adverse effect profile) has been found to be 4 mg^32^ and the optimal effective dose in patients with schizophrenia is 6 mg.^33^

Literature reviews show a wide variation of the prevalence of HDAP from 15% to 41%. Barbui et al. in their outpatient schizophrenia cohort in four European countries reported a prevalence rate of 28% while using a PDD/DDD ratio greater than 1.5 as the operational criteria.^34^ In the United Kingdom, the prevalence of HDAP obtained in an inpatient audit of United Kingdom hospitals involving 49 mental health services using the BNF % maximum as an operational criteria was 10.4%.^35^ This reportedly low prevalence rate increased to 34% when ‘prn’ prescriptions of antipsychotics were considered in the HDAP determination.^36^ In Asia, the reported prevalence of HDAP appears to be lower than values obtained in other parts of the world.^19,37^ However, most of the Asian studies used CPZEq > 1000 mg as the operational criteria, which could introduce some bias, given the differing methodological considerations used in its determination of dose equivalents for CPZEq and the inaccuracies of CPZEq in dose conversions for atypical antipsychotics. In fact, due to these inconsistencies, the American Psychiatric Association practice guideline on the treatment of schizophrenia did not provide any chlorpromazine dose equivalents to atypical antipsychotics.^38^

Globally, the reported differences in the prevalence rates of HDAP may be because of the differing methodological differences such as type of study population (inpatients or outpatient groups), specificity of diagnostic label used and the preferred operational definition of HDAP used in each study. Most international drug policy guidelines discourage HDAP. For example, the Royal College of Psychiatrists’ consensus document on high-dose medication recommends that any prescription of high-dose antipsychotic treatment should be cautiously seen as a time-bound individual trial with a clearly defined treatment target as documented in the treatment plan, which must be subjected to scheduled clinical evaluation and safety monitoring. They also advised that HDAP should only be sustained if the trial provides evidence that the benefits outweigh the attendant risk of safety and tolerability in the affected individual.^39^ It is also advisable that before a trial of HDAP, all monotherapy options, including the optimisation of clozapine therapy, should have been previously optimised without success. Service users on HDAP trial should be closely monitored by clinical evaluation, vital blood tests and electrocardiogram checks, so that any detected abnormality should alert the clinician to discontinue its use.^39^

### Correlates of high-dose antipsychotic prescribing

Males were significantly more likely to receive HDAPs compared with females. Similar findings were reported by other researchers.^13,37,40^ Some researchers have suggested that a male association with HDAP may reflect clinicians’ preference rather than genuine pharmaco-genetic differences and the propensity for aggression in some males compared with females.^40,41^

The unemployed had nearly two-fold risk of receiving HDAP than the employed. This observed association was in variance with the report of Adesola et al.^10^ and was not evaluated in some European or Asian studies.^13,19,37^ It stands to reason that the burden of unemployment amongst persons with schizophrenia in developed countries may be buffered by unemployment support from government that is sadly not readily available in low- and middle-income countries.

Higher average medication cost was associated with HDAP. Most researchers have not evaluated this association in their study designs, especially for inpatient studies where medication cost may be included in the total cost of admission or by the health insurance scheme.^10,19,42^ However, outpatients on high dose antipsychotic treatment are more likely to be on polypharmacy, especially on typical and atypical antipsychotic combinations,^24,43^ and this could be because of treatment resistance and the need for pharmacotherapeutic augmentation. Also, patients with chronic schizophrenia could be on high-dose regimen owing to tolerance to antipsychotics, requiring increasing dose to produce the expected outcome. Presently in Nigeria, the average cost of atypical antipsychotics is 20–35 times more than the cost of typical antipsychotics,^43,44^ which may be the plausible explanation for the given association.

Longer duration of illness and longer duration of treatment were observed to be significantly associated with high dose prescription. Wilkie et al. had earlier reported that service users with illness duration of more than 5 years were more likely to receive high-dose antipsychotic medications.^45^ Furthermore, Lelliot et al. argued that the length of illness could play a significant role in HDAP, given that schizophrenia is a chronic debilitating illness that runs a relapsing–remitting course and clinicians may increase doses or combine drugs to improve response in poor responders.^35^

Antipsychotic polypharmacy (APP) was strongly associated with HDAP such that participants on APP had very high odds of receiving high-dose antipsychotic treatment as was also reported by other researchers.^10,13,19,35,37^ Barbui et al. found that the persistence of high antipsychotic doses from admission to discharge was associated with the simultaneous use of typical and atypical antipsychotics.^34^ Also, the combination of depot antipsychotics with oral medications strongly associated with high-dose antipsychotics beyond the BNF% maximum in outpatients with schizophrenia.^21^ The given evidence suggests that typical antipsychotic depot preparations, either singly or in combination, may greatly increase the odds of HDAP.^46^

Greater severity of psychopathology was found to be strongly associated with high dose prescribing as higher mean PANSS total score and higher mean scores for the subscales were observed amongst participants on HDAP. This finding has been replicated by other researchers, although previous evidence seem to be limited to the PANSS positive subscale.^13,19,47^ On the contrary, Barbui et al. reported that the BPRS ‘negative’ scores were protective against high dose prescribing.^13^ This observed difference may be related to the higher rate of prescription of generic atypical antipsychotics in the developed countries. Further tests may be required to clarify this association.

Participants receiving HDAP were significantly more likely to report greater side effects almost on all domains. This reinforces the firm stance of the Royal College of Psychiatrists’ consensus document against the unchecked practice of HDAP because of the overwhelming evidence of greater side effect burden.^39^ In a meta-analysis of 22 studies by Bolloni et al., HDAP not only failed to produce marked clinical improvements when compared with average doses but also led to an increase in the frequency and severity of adverse effects.^48^

Furthermore, HDAP has also been associated with marked sedation, chronic constipation, impaired cognition, weight gain and hyperprolactinaemia.^49,50,51^

Respondents on a HDAP were less likely to adhere to their medications. Possible reason could be that those patients on HDAP who experienced symptom relief may stop taking their prescribed medications on the presumption of full recovery from the illness. Evidence suggests that covert non-adherence by service users may be inadvertently perceived as poor drug effectiveness by clinicians, which in turn implores the clinician to either prescribe higher doses or add a secondary medication.^52^ One of the management options clinicians adopt to combat medication non-concordance is the use of long-acting injectable antipsychotics. This is especially seen in oral and depot antipsychotic combinations as reported by research.^43^ Also, with poor adherence comes the increased risk of relapse, re-hospitalisation, suicide attempts, a reduction in the overall quality of life and ultimately, poor prognosis.^53,54^ High dose antipsychotic prescription often involves multiple medications and a shorter dosing interval, which could worsen adherence in patients with schizophrenia.^55^

### Predictors of high dose prescribing

Antipsychotic polypharmacy (APP) and anticholinergic use were independent predictors of HDAP as was confirmed by previous research works.^10,13,19,35,36,42^ Paton et al. reported that patients on APP were 20 times and more likely to be on HDAP while Hung et al. also reported an aOR of 8.8 and 10.82 in inpatient and outpatient populations, respectively.^36,37^ Therefore, APP is the strongest predictor of high dose prescribing. Any reduction of HDAP should endeavour to lower the associated combination polytherapy.

Concomitant anticholinergic prescription also increased the odds of HDAP by a three-fold increase. Similar findings have been reported by other researchers.^10,56^ Evidence suggests that HDAP may cumulatively increase the side effect burden on patients, making it necessary for the prophylactic use of anticholinergics, despite the arguments that surround the prophylactic use of anticholinergic agents in patients with schizophrenia.^41,56^ In view of the given discussion, the Schizophrenia Patient Outcomes Research Team (PORT) recommended that the concomitant anticholinergic prophylaxis prescription should be individualised, in which the patient preferences, past history of EPSEs and the attendant side effects of anticholinergic agents such as worsening of cognitive deficits, euphoria, exacerbation of psychosis and other peripheral effects be considered in the decision-making process.^56,57,58^ More so, it could be rewarding to notice that some service users are able to safely and completely discontinue anticholinergic prophylaxis.^59^

Furthermore, experiencing a current psychotic episode was a predictor of high-dose antipsychotic prescribing. This is in line with research evidence which suggests that in relapsed psychotic states, some patients may require higher doses of antipsychotics to achieve the required response, resulting in HDAP in those who have a current psychotic episode.^60,61^

## Strengths and limitations

The strength of our study is that this is the first comprehensive study of HDAP amongst outpatients with schizophrenia in Nigeria. Also, the use of a specific diagnostic group in our study eliminated the probability of group bias. Our limitations were; firstly, the single centre nature of the study may limit the generalisation of the results; secondly, the role of prescribers in HDAP was not evaluated; and thirdly, in-patients were not included in our study, thereby making comparisons difficult with similar research works.

## Conclusion

In conclusion, our study showed that HDAP is prevalent especially amongst patients with schizophrenia. Greater illness severity, higher side effect burden and poor medication adherence were significantly associated with high-dose antipsychotic prescribing. Furthermore, APP was a significant predictor of HDAP. We recommend that, during evaluation of patients on high-dose antipsychotics treatment, clinicians should routinely assess for side effects and medication adherence as it may affect overall clinical outcome. Further research, with a cohort or longitudinal design, is desirable to establish causal effects for the identified correlates of HDAP from our study.
